# Oral Focal Mucinosis

**DOI:** 10.34172/aim.31273

**Published:** 2025-02-01

**Authors:** Varsha Vimal Kumar, Savita Jangal Krishanappa, Girish Hemdal Channabasaviah, Mamata Sharad Kamat, Smitha Gowdara Prakash

**Affiliations:** ^1^Department of Oral Pathology & Microbiology, Rajarajeshwari Dental College and Hospital, Bengaluru, Karnataka, India; ^2^Department of Oral Pathology & Microbiology, Bharati Vidyapeeth (Deemed to be University) Pune, Dental College and Hospital, Sangli, Maharashtra, India; ^3^Department of Oral Pathology and Microbiology, Sharavathi Dental College, Shivamogga, Karanataka, India

**Figure 1:**
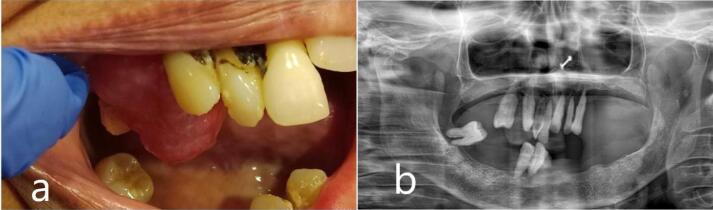
Clinical Presentation and Radiographic Presentation. (a) Pre-operative intraoral photograph revealed a smooth-surfaced, well-defined mass with pinkish hue measuring 2 x2 cm in the 16-tooth region. (b) Orthopantomogram revealed no noticeable alterations associated with growth

 A 65-year-old woman reported a progressive growth in the upper right alveolar region over the past 7 months as her primary concern. Intraoral examination revealed a 2 × 2 cm, smooth-surfaced, non-tender, firm well-defined mass with a pinkish hue in the 16-tooth region (Figure 1a).

**Figure 2:**
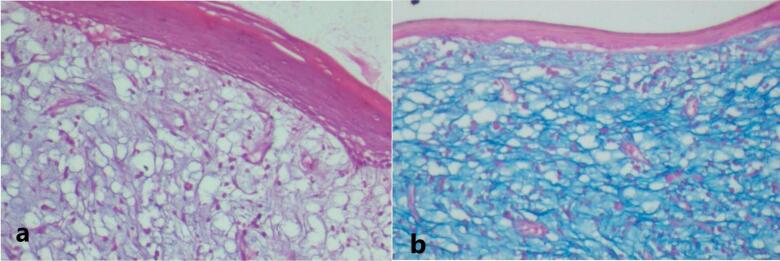
Histopathological Examination. (a) Lesion shows loose fibro-myxoid connective tissue stroma areas, accompanied by an overlying layer of stratified squamous epithelium. (20X, H&E stain). (b) Photomicrograph shows presence of hyaluronic acid by positive alcian blue staining (pH 2.5) in the myxoid stroma (20X)

**Figure 3:**
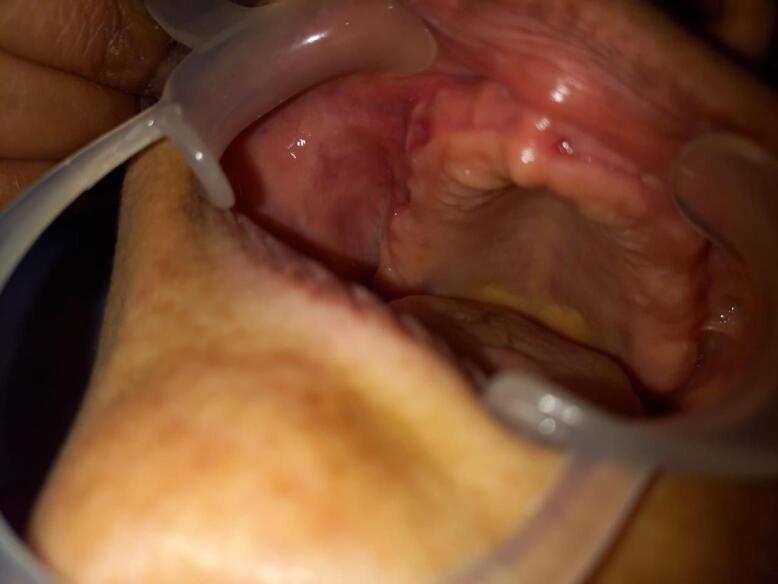
Postoperative Images Depicting the Surgical Site

 The patient exhibited poor oral hygiene, accompanied by halitosis and severely compromised periodontal conditions in some teeth. Medical or dental history had no contributing factors to the diagnosis, and also no noticeable changes were observed on the orthopantomogram related to the growth (Figure 1b), Based on clinical findings, it was diagnosed clinically as pyogenic granuloma following which the mass was completely excised under local anaesthesia and subjected for histopathological diagnosis. Macroscopy revealed a soft to moderately firm whitish-grey mass. Histopathological exploration revealed a connective tissue lesion which was myxomatous in nature and well encapsulated. The lesional tissue was characterized by spindle-shaped fibroblasts interspersed with short collagen bundles (Figure 2a). Myxoid regions stained strongly with Alcian blue, demonstrating the presence of hyaluronic acid whereas the dense connective tissue areas were negative for the same (Figure 2b). The lesional tissue was negative for Reticulin staining. The patient was monitored weekly during the first month, and every 15 days over six months. During follow-up visits, healing was evaluated by measuring wound dimensions, which progressively decreased with each visit. Satisfactory healing was observed as indicated by the absence of infection, redness, or swelling, and there were no signs of recurrence during the six-month follow-up period (Figure 3).

 Oral focal mucinosis (OFM) is a benign, soft-tissue oral lesion which is relatively rare and asymptomatic. It is frequently described as the oral counterpart of cutaneous focal mucinosis.^1^ Tomich *et al.* in 1974coined the term “oral focal mucinosis”.^2^

 The precise origin of OFM remains elusive. While some research indicates that fibroblasts lead to increased production of hyaluronic acid, uncertainty exists/prevails pertaining to the triggering factors causing this overproduction. Although local trauma, local irritation, and masticatory trauma have been proposed as potential causative factors, their role in the development of OFM remains uncertain. It predominantly affects women with a male-female ratio of 1:2.1, in the fourth-fifth decade of life. Intraorally, the most common sites in descending order are the gingiva (58.2%), palate (15.3%), alveolar ridge mucosa (8.2%), buccal mucosa (7.1%) and tongue (6.1%).^3^ The lesion clinically presents as a sessile, painless, nodular mass, blending seamlessly with the surrounding mucosa in color. Its size ranges from a few millimeters to up to 2 cm in diameter.^4^

 OFM manifests as a non-encapsulated, well-defined submucosal mass comprising of extremely loose or myxomatous, or mucinous connective tissue. Within these mucinous regions, fibroblasts are observed in minimal to moderate quantities, often exhibiting delicate, fibrillar processes. Notably, the mucinous zone displays reduced vascularity compared to the surrounding connective tissue, and inflammatory cells are notably absent.^5^ OFM may mimic other oral lesions or pathologies like oral fibroma, myxoid fibroma, soft-tissue myxoma, nerve sheath myxoma, mucous retention phenomenon, and fibrous hyperplasia.^1,6^ The absence of reticular fibers and the sharp demarcation differentiate OFM from soft-tissue myxomas, odontogenic myxoma, and myxomatous changes in fibrous lesions. Additionally, odontogenic myxoma shows the presence of odontogenic rests. In mucous retention phenomena, the lesion is enclosed by either a granulation tissue wall or an epithelium-lined wall, with the mucoid material containing histiocytic cells. These characteristics, however, are not present in OFM.^1-6^ Special staining may be required to arrive at the diagnosis. Management typically involves conservative surgical removal, with a low recurrence rate. Early recognition and proper diagnosis are crucial due to its potential to mimic other oral pathologies.^5^
